# Manic episode, aggressive behavior and poor insight are significantly associated with involuntary admission in patients with bipolar disorders

**DOI:** 10.7717/peerj.7339

**Published:** 2019-07-19

**Authors:** Chenyuli Luo, Hui Chen, Shaoling Zhong, Huijuan Guo, Qiguang Li, Weixiong Cai, Giovanni de Girolamo, Jiansong Zhou, Xiaoping Wang

**Affiliations:** 1 Department of Psychiatry, The Second Xiangya Hospital, Central South University, Changsha, Hunan, China; 2 National Clinical Research Center on Mental Disorders, Changsha, Hunan, China; 3 National Technology Institute on Mental Disorders, Changsha, Hunan, China; 4 Hunan Key Laboratory of Psychiatry and Mental Health, Changsha, Hunan, China; 5 Shanghai Forensic Service Platform, Academy of Forensic Science, Shanghai Key Laboratory of Forensic Medicine, Shanghai, China; 6 IRCCS Istituto Centro San Giovanni di Dio Fatebenefratelli, Brescia, Italy

**Keywords:** Involuntary admission, Bipolar disorder, Aggressive behavior

## Abstract

**Objective(s):**

Serious mental illnesses, such as bipolar disorders and schizophrenia, are closely associated with involuntary admission. Many studies have focused on involuntary admission in people with schizophrenia, but little is known about the factors associated with involuntary admission in Chinese patients with bipolar disorders. This study aimed to investigate socio-demographic and clinical factors associated with involuntary admission in Chinese patients with bipolar disorders.

**Methods:**

In this multi-center cross-sectional survey in China, a total of 155 newly admitted patients with bipolar disorders were consecutively recruited from 16 psychiatric institutions from 15 March to 14 April, 2013. Patients’ socio-demographic and clinical data were collected from their medical records. The Modified Overt Aggression Scale and the Insight and Treatment Attitudes Questionnaire were used to measure patients’ level of aggression and insight of current psychiatric illness.

**Results:**

The prevalence of involuntary admission was 52% in this sample of Chinese inpatients with bipolar disorders. In multiple logistic regression, a high level of aggression (odds ratio (OR) = 2.48), diagnosis of manic episode (OR = 3.65), poor insight (OR = 7.52), and a low level of education (OR = 3.13) were significantly associated with involuntary admission.

**Conclusion:**

Manic episode, aggressive behavior, and poor insight were the significant contributing factors to involuntary admission in Chinese patients with bipolar disorders.

## Introduction

Involuntary admission is a common and controversial issue in psychiatric clinical practice and may raise clinical, ethical, and legal concerns (Hoffmann et al., 2017). It is an act that may violate basic human rights, may have a potential negative effect on patients’ satisfaction and is receiving increased attention in many countries (Iversen, Hoyer & Sexton, 2007; Wang et al., 2015). In Europe, a multicenter prospective study (the “EUNOMIA project”) with 13 psychiatric institutions in 12 European countries has been carried out to investigate involuntary treatment and its influencing factor and outcomes (Giacco et al., 2012; Kalisova et al., 2014; Nawka et al., 2013). The study found that the percentages of psychiatric patients receiving involuntary treatment ranged from 21% to 59%, and aggression against others was the most frequent reason for patients being prescribed involuntary treatment. In another study with a large sample of 2,030 involuntary hospitalized psychiatric patients in 10 European countries, 770 patients (38%) were prescribed coercive measures (Raboch et al., 2010). In Switzerland, the involuntary admission rate among all inpatients aged 18–70 (*N* = 9,698) was 24.8% in 2007 (Lay, Nordt & Rössler, 2011). In a multi-center study of patients who were acutely admitted to psychiatric wards in Norway, 44% were involuntary hospitalized (Hustoft et al., 2013). By contrast, in some countries, such as Italy, the percentage of involuntary patients among all patients in general psychiatric wards does not exceed 9% (De Girolamo et al., 2008). The above data indicate that involuntary admission is common in psychiatric clinical practice, however, the rates vary widely across countries.

In China, some improvements in the protection of psychiatric patients’ rights have been achieved. For example, a multi-center study in 2003 showed that involuntary hospitalization rate in Chinese psychiatric institutions was as high as 82.5% (Pan & Xie, 2003), whereas, 10 years later, the rate of involuntary admission decreased to 42.0–53.1% (Gou et al., 2014; Zhou et al., 2015). However, compared to the above-mentioned rates of involuntary admission in Europe, these is still much space for reducing the rate of involuntary admission in China.

Involuntary treatment is associated with many negative clinical outcomes. Obviously, involuntary patients are less likely to adhere to treatment (Craw & Compton, 2006). Compared to voluntary patients, involuntary patients have higher suicide rates and lower levels of social functioning (Kallert, Glockner & Schutzwohl, 2008), which may raise medical disputes and will lead to poor therapeutic relationship (Sheehan & Burns, 2011). In addition, a commonly perceived concern is the poor therapeutic relationship between patients and clinicians and poor treatment satisfaction resulted from involuntary hospitalization (Sheehan & Burns, 2011; Katsakou et al., 2010; Georgieva et al., 2017).

Involuntary admission in psychiatric patients is affected by many factors, including demographics, symptoms, and the legal system of the country (Fiorillo et al., 2011). Many studies have shown that high level may reduce the risk of involuntary admission (Hustoft et al., 2013; Zhou et al., 2015). Aggressive behavior and positive symptoms are associated with a high likelihood of involuntary admission in Western countries (Fiorillo et al., 2012; Hustoft et al., 2013; Van Der Post et al., 2009). In our previous study, poor social functioning and insight were also found to be related to psychiatric patients involuntary admission (Gou et al., 2014).

Schizophrenia and bipolar disorders are the most common psychiatric diagnoses associated with involuntary admission. In Germany, bipolar disorders and schizophrenia account for 18.9% and 30.5% of the total number of involuntary admissions, respectively (Hoffmann et al., 2017). In our previous study, we reported that male gender, history of hospitalization, aggressive behavior, and diagnosis of schizophrenia or related disorders were the risk factors of involuntary admission in China (Zhou et al., 2015). In Ireland, a study with samples of patients with schizophrenia and bipolar disorders found that increased age was associated with involuntary admission in patients with bipolar disorders but not in patients with schizophrenia (Kelly et al., 2018). Nevertheless, very few studies have specifically investigated factors associated with involuntary admission of patients with bipolar disorders. Given the different clinical features between bipolar disorders and schizophrenia, we speculate that factors affecting involuntary admission in patients with bipolar disorders might be quite different from those in patients with schizophrenia.

Anyway, under any circumstance, involuntary admission should be minimized as much as possible to maximize patients’ benefits (Fiorillo et al., 2012). In the Article 30 of the China Mental Health Law (Zhao & Dawson, 2014), the criteria for involuntary admission are “*psychiatric admissions and treatment should be determined solely by the patient, unless he or she is evaluated as having a severe mental disorder and has self-harmed or is at risk of self-harming behavior or has harmed others or is at risk of harming others*” (Zhou et al., 2015). Thus, we hypothesized that poor of insight, aggression, suicidal history, and some clinical characteristics of bipolar disorders (e.g., manic or depressive episodes) may be related to involuntary admission among patients with bipolar disorders. As there are many differences in healthcare systems, cultural environments and legal systems between China and Western countries (Zhou et al., 2015; Zhong et al., 2017), it would be interesting to investigate factors associated with involuntary admission in patients with serious mental illnesses in China. The present study was carried out to examine factors associated with involuntary admission in Chinese patients with bipolar disorders.

## Materials and Methods

### Study sites and patients

This study was a multi-center cross-sectional survey, which was sponsored by the Chinese Psychiatric Association. The data were collected from 16 representative psychiatric institutions in China between 15 March and 14 April 2013. Details of these facilities have been described in a previously published paper (Zhou et al., 2015). In brief, all patients with bipolar disorders who were admitted to the 16 participating facilities during an index month were consecutively invited to participate in the study. Patients who were aged 18 years or older and met ICD-10 diagnostic criteria for bipolar disorders were deemed eligible for this study. We excluded patients with history of brain trauma and current substance use disorders.

The informed consent was obtained from all participants and their guardians. The study was approved by the Biomedical Ethics Board of the Second Xiangya Hospital, Central South University in Hunan Province (2013068) and by the ethics committees of all participating institutions.

### Instruments and evaluation

A standardized form was designed to collect data from inpatients’ medical records, including socio-demographics (e.g., age, gender, residence place, education, and employment status) and clinical characteristics (e.g., insight, suicide history, age of onset, and length of illness) (Gou et al., 2014). Following ICD-10 criteria, the diagnosis of bipolar disorders was categorized into two groups: manic and depressive episodes.

The Chinese Modified Overt Aggression Scale (MOAS) (He et al., 2017; Yudofsky et al., 1986) was used to assess aggressive and violent behavior in the week before admission. Information was collected directly from patients, their family members, and medical records (Zhou et al., 2015). The Chinese MOAS has been proved to be reliable and valid for assessing aggressive and violent behavior of psychiatric patients (Xie & Zheng, 2001). The MOAS includes four subdomains of aggression: verbal, against objects, against self, and physical-interpersonal. The score of each subdomain ranges from 0 to 4 with a higher score indicating more severe aggressive behavior. The total MOAS score was calculated according to the following formula: 1 × verbal score + 2 × against objects score + 3 × against self score + 4 × physical-interpersonal score. The total weighted score for each evaluation ranges from 0 (no aggression) to 40 (maximum grade of aggression). Based on previous study (Zhou et al., 2016), the MOAS total score 5 or higher was assigned high aggressive, 4 of lower was assigned low aggressive.

The Chinese Insight and Treatment Attitudes Questionnaire (ITAQ) was used to assess patients’ insight (McEvoy et al., 1989; Liu et al., 1995). The ITAQ comprises of 11 items, and all items were rated on a 3-point scale from 0 to 2. The total score ranges from 0 to 22 with a higher score indicating a better insight.

In China, most patient admissions were decided by their family members or the treating clinician. Thus, on the basis of our previous study (Zhou et al., 2015), we determined the type of admission according to patients’ self-report, by asking: “Did you voluntarily go to hospital?” and “Were you forced by your family or others?” Patients who denied the first question or endorsed the second question were classified as having involuntary admission. The patients were interviewed within one week before discharged from hospital. Data on scores of MOAS and ITAQ were directly copied from the medical records.

### Statistical analysis

Analysis was conducted with SPSS 19.0 for Windows statistical package. Independent sample *t*-test and Chi-square test were used to compare the socio-demographic and clinical characteristics of patients who were admitted voluntarily and involuntarily as appropriate. For further analysis, we used the median split approach to dichotomize the ITAQ total score into poor insight (ITAQ ≤ 17) and good insight (ITAQ > 17). Multiple binary logistic regression model (Backward: LR) was used to identify factors associated with involuntary admission. The associations between factors and involuntary admission were quantified with odds ratios (ORs) and their 95% confidence intervals (CIs). The level of statistical significance was set at 0.05 (two-tailed).

## Results

### Characteristics of the study sample and prevalence of involuntary admission

**Figure 1:**
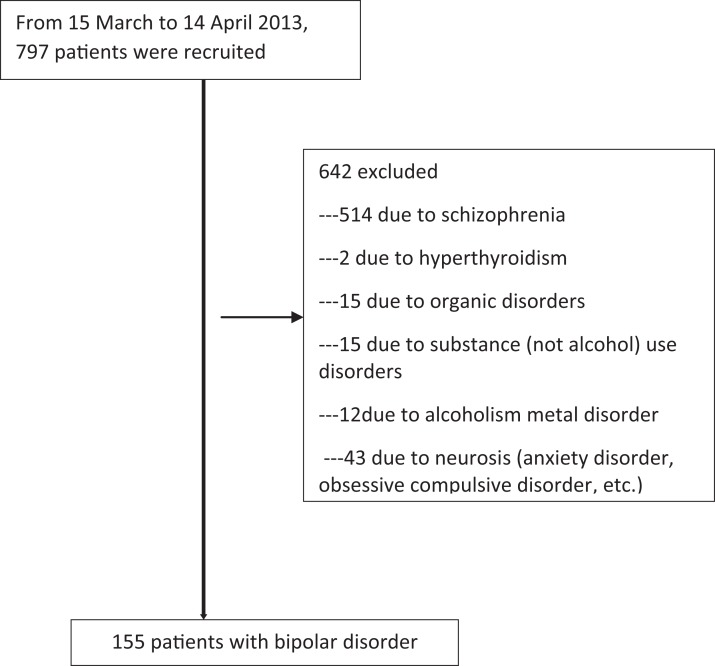
Flow-chart showing sample recruitment.

Figure 1 shows a flowchart of the patients’ recruitment process. In the index month, a total of 155 patients with bipolar disorders successfully completed the study, consisting of 74 (47.7%) voluntary and 81 (52.3%) involuntary admissions. Among these participants, 60 (38.7%) were males, 81 (52.3%) were in a manic episode, and 57 (36.8%) were rated as having no insight.

### Comparison of demographic and clinical characteristics between voluntarily and involuntarily admitted patients

As shown in Table 1, compared to voluntarily admitted patients, involuntarily patients were more likely to have low level of education (χ^2^ = 3.49, d*f* = 1, *p* = 0.06), be unemployed (χ^2^ = 48.8, d*f* = 1, *p* < 0.001), have longer duration of illness (*t* = −2.5, d*f* = 133, *p* = 0.050), have a past history of hospitalization (χ^2^ = 4.6, d*f* = 1, *p* = 0.030), have manic episode (χ^2^ = 11.8, d*f* = 1, *p* = 0.001), have poor insight (χ^2^ = 14.2, d*f* = 2, *p* = 0.001), and have a higher aggression score (τ = −3.0, d*f* = 153, *p* < 0.001) and a lower ITAQ score (τ = 5.8, d*f* = 153, *p* < 0.001).

**Table 1:** Sociodemographic and clinical characteristics of the sample.

Variables	Voluntary (*N* = 74)		Involuntary (*N* = 81)		Statistics		
	*N*	%	*N*	%	χ^2^	d*f*	*p*
Gender					0.05	1	0.83
Male	28	37.8	32	39.5			
Female	46	62.2	49	60.5			
Employ					48.8	1	<0.001
Employed	53	71.6	13	16.0			
Unemployed	21	28.4	68	84.0			
Marital status					0.41	1	0.52
Unmarried	44	59.5	44	54.3			
Married	30	40.5	37	45.7			
Education					3.49	1	0.06
Low (≤9 years)	30	40.5	45	55.6			
High (>9 years)	44	59.5	36	44.4			
Residence					0.1	1	0.71
Rural	28	37.8	33	40.7			
Urban	46	62.2	47	59.3			
Health insurance					1.65	1	0.20
Yes	56	75.7	68	84.0			
No	18	24.3	13	16.0			
Outpatient treatment prior to index admission					0.03	1	0.86
Yes	49	66.2	57	70.4			
No	25	33.8	24	29.6			
Diagnosis					11.8	1	0.001
Manic episode	28	37.8	53	65.4			
Depressive episode	46	62.2	28	34.6			
Insight					20.6	1	<0.001
Poor insight (ITAQ ≤17)	18	24.3	49	60.5			
Good insight (ITAQ >17)	56	75.7	32	39.5			
Previously hospitalized					4.6	1	0.03
Yes	48	64.9	65	80.2			
No	26	35.1	16	19.8			
History of violence					1.5	1	0.22
Yes	7	9.5	13	16.0			
No	67	90.5	68	84.0			
History of self-harm					0.0	1	0.99
Yes	10	13.5	11	13.6			
No	64	86.5	70	86.5			
Aggrssive					8.5	1	0.01
Low (MOAS ≤4)	51	68.9	37	45.7			
High (MOAS ≥5)	23	31.1	44	54.3			
	**Mean**	**SD**	**Mean**	**SD**	**τ**	**d*f***	***p***
Age	38.2	15.9	38.5	14.8	−0.1	153	0.89
Age of onset (years)	33.8	15.2	30.7	13.8	1.3	150	0.20
Length of illness (month)	56.0	69.3	94.2	115.3	−2.5	133	0.05
MOAS	4.1	5.8	7.2	6.9	−3.0	153	<0.001
ITAQ	19.3	3.5	14.7	5.8	5.8	153	<0.001

**Note:**

MOAS, Modified Overt Aggression Scale; ITAQ, the Insight and Treatment Attitudes Questionnaire.

### Factors associated with involuntary admission in patients with bipolar disorders

As shown in Table 2, multiple binary logistic regression analysis revealed that variables significantly associated with involuntary admission were manic episode (OR = 3.65, 95% CI [1.65–8.09]), poor insight (OR = 7.52, 95% CI [3.17–17.85]), high aggressive (OR = 2.48, 95% CI [1.14–5.40]), and low level of education (OR = 3.13, 95% CI [1.37–7.17]).

**Table 2:** Independent contributors to involuntary admission patients with bipolar disorder (binary logistic regression model).

	Involuntary admission		
	Odds ratio	95% CI	*p*-value
Diagnosis (Manic episode)	3.65	[1.65–8.09]	0.001
Poor insight	7.52	[3.17–17.85]	<0.001
Low level of education	3.13	[1.37–7.17]	0.007
High aggressive	2.48	[1.14–5.40]	0.022

**Note:**

The study site has been controlled for as a covariate.

## Discussion

To the best of our knowledge, this is the first study to examine the involuntary admission of patients with bipolar disorders in China. Our study found that 52.3% of the Chinese inpatients with bipolar disorders were involuntary patients, and involuntary admission were significantly associated with manic episode, poor insight, more severe aggression and a low level of education.

The 52% rate of involuntary admissions in Chinese patients with bipolar disorder is higher than the 42% rate in all psychiatric inpatients from our previous survey (42.0%) in China (Zhou et al., 2015) and also higher than those of patients with bipolar disorders in many Western countries, such as Germany (18.9%) (Hoffmann et al., 2017). However, the risk factors for involuntary admissions are consistent with the findings found in previous studies in Western countries (Hustoft et al., 2013; Correll et al., 2017; Coid et al., 2018). In the present study, we found that low education level was associated with an increased risk of involuntary admission. Patients with a high level of education may have good comprehension and coping skills (Zhang, Harvey & Andrew, 2011), which can help patients completely understand their mental health status, communicate effectively with clinicians, and accept the need for hospitalization. Because of this, patients with a low level of education are more likely to be admitted involuntarily.

Our results suggest that manic episodes are more likely to lead to involuntary hospital admission than depressive episodes. This feature of involuntary hospitalization for bipolar disorders is consistent with our clinical experience. In general, patients in a manic episode are highly likely to be excitable and display psychotic symptoms, even psychomotor excitement and agitation, resulting in violent behaviors (Coid et al., 2018). Patients with these disturbed and disturbing behaviors are highly likely to be forced into the hospital. In general, patients in a depressive episode are at higher risk of suicide or self-harm behavior, which would result in involuntary hospitalization. However, we did not find the significant association between involuntary admission and a past history of suicidal behaviors; this finding is in line with a recent study (Stylianidis et al., 2017), showing that depression was a protective factor for involuntary admission. One possible explanation that would be depressed patients have relatively good insight compared to manic patients, and they are more likely to actively seek treatment (Raveesh et al., 2016).

As expected, we found more severe aggression was significantly associated with involuntary admission in patients with bipolar disorders. Because severe aggression is presented with acute agitation and dangerous behaviors, this association is plausible and keeping with findings from previous studies with samples of patients with schizophrenia (Correll et al., 2017; San et al., 2016).

Insight is a multidimensional concept that includes awareness of having psychopathological symptoms, the need for and acceptance of treatment, and awareness for the social consequence of that disorder (Gou et al., 2014). In general, a patient with good insight will voluntarily seek treatment and may have good compliance and benign drug-attitude (Ramachandran et al., 2016; Schuepbach et al., 2006). In the current study, poor insight was associated with a high risk of involuntary admission in patients with bipolar disorders, replicating the frequently reported association between poor insight and involuntary admission in patients with schizophrenia spectrum disorders (Montemagni et al., 2011).

### Limitations

This study has some limitations. First, the definition of involuntary admission was based on patients’ self-report rather than on the legal status of each admission (voluntary/involuntary). For this reason, directly comparing our results with those of Western studies might be difficult. Second, some factors that may also affect the type of admission of patients with bipolar disorders, such as the season of the onset, social support, and stigma, were not investigated in this study. Finally, probably due to the relatively small sample of patients with bipolar disorders, our study failed to find an association between history of suicidal behaviors and involuntary admission. Studies with a large sample of patients with bipolar disorders are warranted to clarify the relationship between history of suicidal behaviors and involuntary admission.

## Conclusions

Involuntary admission and hospital treatment have been widely accepted as indispensable measures to protect patients, others, and society; however, this situation still raises many problems (Zhang et al., 2015). Reducing the rate of involuntary admission and ensuring that patients with mental disorders receive appropriate treatments need further investigation. In this study, 52% of the Chinese patients with bipolar disorders were admitted involuntarily, and factors associated with involuntary admission in the context of bipolar disorders are somehow different from those found in patients with schizophrenia. This difference highlights the possible impact of the specific characteristics of each disorder on admission patterns. The results of our study may help develop programs for preventing and reducing involuntary hospitalizations for bipolar disorders and may help set up additional humane and friendly mental health services.

## Supplemental Information

10.7717/peerj.7339/supp-1 Supplemental Information 1 Raw data.

10.7717/peerj.7339/supp-2 Supplemental Information 2 Codebook.

## Additional Information and Declarations

### Data Availability

The following information was supplied regarding data availability: The raw measurements are available in File S1.
