# Supplementary material for: Manic episode, aggressive behavior and poor insight are significantly associated with involuntary admission in patients with bipolar disorders
Source: PeerJ. 2019 Jul 19;7:e7339. doi: 10.7717/peerj.7339 (PMC6644629; doi:10.7717/peerj.7339)
Supplement: Supplemental Information 2 [file peerj-07-7339-s002.doc]

Codebook

AGE: years

SEX: 1=male, 2=female

[Course](javascript:;): [course](javascript:;) [of](javascript:;) [disease](javascript:;) (months)

FIRSTAGE: Age of first onset (years)

Insight: 1=Good insight, 2=Poor insight, 3=Lack of insight

diagnose: 1=Depressive episode, 2= Manic episode

admission: 1=voluntary, 2=involuntary

marriage: 1=unmarried, 2=married

education: years of education

residence: 1=urban; 2=Rural residence

occupation: 1= Unemployed, 2= employed

admssion1: (admission history) 1=none, 2=yes

aggression: (aggression or violence history) 1=none, 2=yes

suicide: (suicide history) 1=none, 2=yes

MOAS: total of MOAS score
